# Body-Plan Reorganization in a Sponge Correlates with Microbiome Change

**DOI:** 10.1093/molbev/msad138

**Published:** 2023-06-08

**Authors:** Sergio Vargas, Laura Leiva, Michael Eitel, Franziska Curdt, Sven Rohde, Christopher Arnold, Michael Nickel, Peter Schupp, William D Orsi, Maja Adamska, Gert Wörheide

**Affiliations:** Department of Earth and Environmental Sciences, Palaeontology & Geobiology, Ludwig-Maximilians-Universität München, München, Germany; Department of Earth and Environmental Sciences, Palaeontology & Geobiology, Ludwig-Maximilians-Universität München, München, Germany; Department of Earth and Environmental Sciences, Palaeontology & Geobiology, Ludwig-Maximilians-Universität München, München, Germany; Department of Environmental Biochemistry, Institute for Chemistry and Biology of the Marine Environment Terramare, Carl-von-Ossietzky University Oldenburg, Wilhemshaven, Germany; Department of Environmental Biochemistry, Institute for Chemistry and Biology of the Marine Environment Terramare, Carl-von-Ossietzky University Oldenburg, Wilhemshaven, Germany; Institut für Spezielle Zoologie und Evolutionsbiologie, Friedrich-Schiller-Universität, Jena, Germany; Institut für Spezielle Zoologie und Evolutionsbiologie, Friedrich-Schiller-Universität, Jena, Germany; Department of Environmental Biochemistry, Institute for Chemistry and Biology of the Marine Environment Terramare, Carl-von-Ossietzky University Oldenburg, Wilhemshaven, Germany; Helmholtz Institute for Functional Marine Biodiversity, Carl-von-Ossietzky University Oldenburg, Wilhemshaven, Germany; Department of Earth and Environmental Sciences, Palaeontology & Geobiology, Ludwig-Maximilians-Universität München, München, Germany; GeoBio-Center, Ludwig-Maximilians-Universität München, München, Germany; Research School of Biology, Australian National University, Canberra, Australia; Department of Earth and Environmental Sciences, Palaeontology & Geobiology, Ludwig-Maximilians-Universität München, München, Germany; GeoBio-Center, Ludwig-Maximilians-Universität München, München, Germany; SNSB-Bayerische Staatssammlung für Paläontologie und Geologie, München, Germany

**Keywords:** microbiome, sponges, holobiont, symbiosis

## Abstract

Mounting evidence suggests that animals and their associated bacteria interact via intricate molecular mechanisms, and it is hypothesized that disturbances to the microbiome influence animal development. Here, we show that the loss of a key photosymbiont (i.e., bleaching) upon shading correlates with a stark body-plan reorganization in the common aquarium cyanosponge *Lendenfeldia chondrodes*. The morphological changes observed in shaded sponges include the development of a thread-like morphology that contrasts with the flattened, foliose morphology of control specimens. The microanatomy of shaded sponges markedly differed from that of control sponges, with shaded specimens lacking a well-developed cortex and choanosome. Also, the palisade of polyvacuolar gland-like cells typical in control specimens was absent in shaded sponges. The morphological changes observed in shaded specimens are coupled with broad transcriptomic changes and include the modulation of signaling pathways involved in animal morphogenesis and immune response, such as the Wnt, transforming growth factor *β* (TGF-*β*), and TLR–ILR pathways. This study provides a genetic, physiological, and morphological assessment of the effect of microbiome changes on sponge postembryonic development and homeostasis. The correlated response of the sponge host to the collapse of the population of symbiotic cyanobacteria provides evidence for a coupling between the sponge transcriptomic state and the state of its microbiome. This coupling suggests that the ability of animals to interact with their microbiomes and respond to microbiome perturbations has deep evolutionary origins in this group.

## Introduction

The microbiome can alter numerous host physiological and developmental processes in different bilaterian animal systems. For instance, the renewal of the intestinal epithelium in zebrafish is co-regulated by signals from its microbiome (Cheesman et al. 2011). Brain development in mice also appears to be modulated by the gut microbiota, and evidence exists for a role of the microbiome on mouse behavior (Heijtz et al. 2011; Sampson and Mazmanian 2015; Sharon et al. 2016). Similarly, in *Drosophila* the microbiome affects the locomotion behavior of the host, modulates its metabolic homeostasis and development via direct signaling, and has direct transgenerational effects on female fitness (Shin et al. 2011; Morimoto et al. 2017; Schretter et al. 2018). Nonbilaterian animals also appear to interact in complex ways with their microbiomes. In the cnidarian *Hydra vulgaris*, for instance, bacterial colonization during the early life stages is mediated by maternally produced antimicrobial peptides and secreted neuropeptides with antibacterial properties regulate the composition of the microbiome of adult *Hydra* individuals, which contraction behavior can be modulated by their associated bacteria (Fraune et al. 2010; Augustin et al. 2017; Klimovich et al. 2017). Thus, host–microbiome interactions appear to have deep evolutionary roots in the animal lineage.

Sponges, one of the two phyla most likely representing the sister group to all remaining animal lineages (King and Rokas 2017), harbor bacterial communities of varying complexity and richness (Thomas et al. 2016) and form holobionts with different degrees of physiological integration (Webster and Thomas 2016). Eukaryotic-like domain-containing proteins putatively used for host interaction are enriched in the genomes of sponge bacterial symbionts (Reynolds and Thomas 2016; Germer et al. 2017; Moeller et al. 2019; Kelly et al. 2022), and sponge genome or (meta-) transcriptome screenings have found a rich set of proteins involved in immunity or pattern recognition in these animals and have suggested a role of these proteins in the regulation of sponge–bacteria symbioses (Riesgo et al. 2014; Yuen et al. 2014; Degnan 2015; Guzman and Conaco 2016). *Amphimedon queenslandica*, for instance, possesses a remarkable set of NLR genes (Degnan 2015), and the calcifying demosponge *Vaceletia crypta* expresses a diverse set of Toll-like receptors and NLR genes (Germer et al. 2017). Studies on sponges found in the field in symbiotic and aposymbiotic states revealed a potential role of scavenger receptor cysteine-rich (SRCR) domain-containing proteins in the interaction between the sponge and its microbiome (Steindler et al. 2007). Scavenger receptors are involved in pattern recognition (Resnick et al. 1994), innate immune response in mammals (Hohenester et al. 1999; Canton et al. 2013), and mediate bacterial phagocytosis (Canton et al. 2013).

Among sponges, cyanosponges (i.e., sponges with cyanobacteria-dominated microbiomes) somewhat represent an extreme level of integration between the sponge host and its microbiome. Cyanosponges can be mixotrophs combining filter-feeding with symbiont-derived nutrition (Hudspith et al. 2022) or autotrophs, in which case their photosynthetic symbionts can provide up to 80% of their carbon requirements (Usher 2008). Field experiments have shown that in some cyanosponges, photosymbiont loss can lead to a 42% reduction in biomass after a 2-week exposure to shading (Thacker 2005) and that the sponge hosts do not compensate for the reduction in symbiont-derived carbon resulting from shading through increased filter-feeding (Freeman and Thacker 2011). These observations indicate a high degree of physiological integration between cyanosponges and their microbiomes. In agreement, field experiments on cyanobacteria-bearing keratose sponges revealed that these sponges change their morphology after losing their cyanobacterial symbionts upon transplantation to deeper (e.g., 200 m) waters, likely to increase their filtering capacity in response to a reduced symbiont-derived carbon input (Maldonado and Young 1998).

**Fig. 1.**
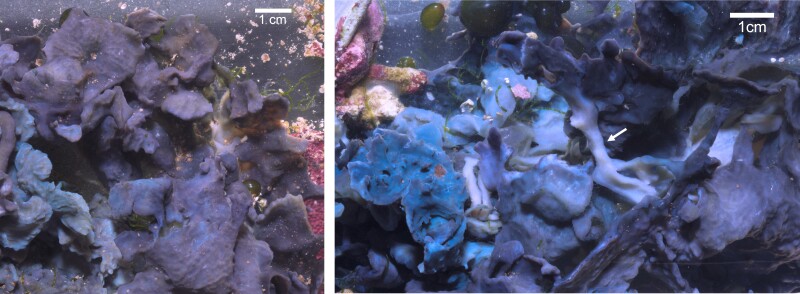
*Lendenfeldia chondrodes* growing under control conditions. Left: sponge viewed from the top; note the flattened, foliose morphology. Right: Bottom of the sponge is shown in the left panel. Pale and elongated sections (white left arrow) connect foliose sections of the sponge.

The observed active response of cyanosponges to the collapse of the photosymbiont population suggests that a compositional reorganization of the sponge microbiome in response to environmental changes can result in a postembryonic developmental reorganization of the sponge body-plan. To study this phenomenon, we use the autotrophic cyanosponge *Lendenfeldia chondrodes*, a common salt-water aquarium sponge (Galitz et al. 2018). Under normal culture conditions, *L. chondrodes* displays two different colorations and growth forms depending on the exposure of the sponge tissues to light (fig. 1). Sections of the sponge exposed to light are purple to dark blue and display a foliose growth form, while shaded parts of the sponge are white and grow to form thread-like projections (fig. 1). Here, we investigate the effect of shading on *L. chondrodes*’ microbiome, host morphology, and host transcriptional state. Shading *L. chondrodes* causes the loss of its cyanobacterial photosymbionts and results in a marked host response. This response is characterized by a reorganization of the sponge body-plan and growth form, and it is accompanied by a broad transcriptional response involving immune and developmental coexpression modules. In conjunction, these results offer support for a mechanistic coupling between the sponge's physiological and transcriptional state and its microbiome, allowing the host to respond to changes in their symbiotic communities triggered by the environment.

## Results

### Shading Results in the Collapse of the Cyanobacterial Population in *L. chondrodes*

**Fig. 2.**
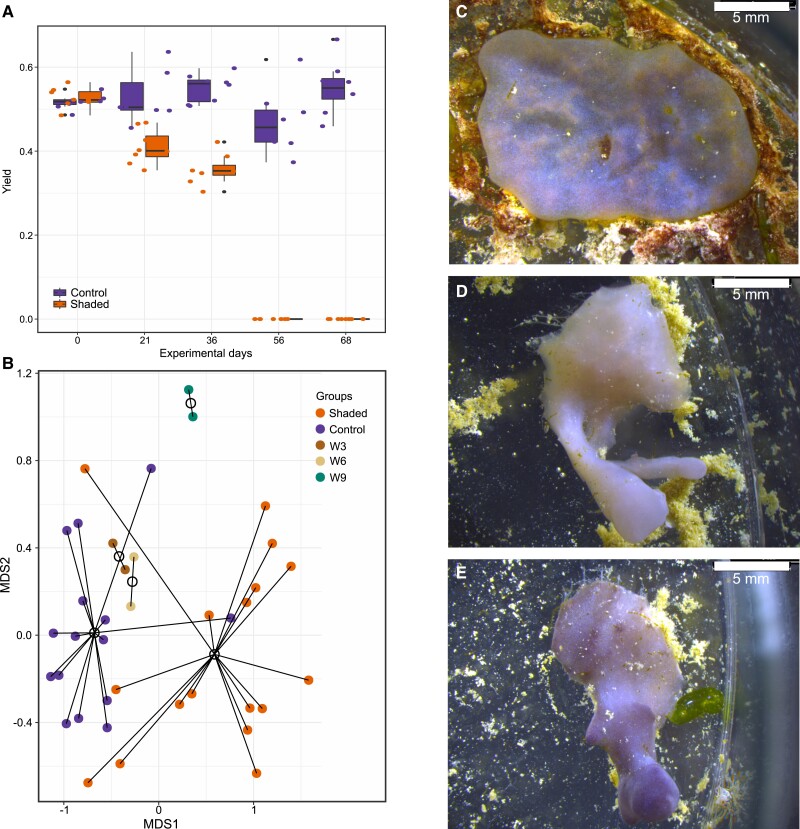
Photosynthetic yield, microbiome composition, and morphological changes observed in control versus shaded *L. chondrodes* explants. (*A*) Photosynthetic yield of sponges in control and shading treatments at different time points during a characteristic shading experiment. (*B*) NMDS (Bray–Curtis dissimilarity) showing the microbiome composition of *L. chondrodes* explants exposed to zero (i.e., control), 3 (W3), 6 (W6), 9 (W9), and at least 12 weeks of shading (i.e., white/bleached). (*C–E*) *L. chondrodes* explants after 12 experimental weeks. Note the development of projections in explants exposed to shading (*D* and *E*) versus control conditions (*C*).

Shading *L. chondrodes* caused a progressive loss of its characteristic blue/purple color (i.e., bleaching) and a drop in *chlorophyll-a* activity (fig. 2*A*). High-throughput 16S V4 rRNA amplicon sequencing of foliose (i.e., blue/purple) and thread-like (i.e., white/bleached) *L. chondrodes* revealed a significant shift of this species’ microbiome during shading (ANOSIM *R*_1000_ = 0.4234, *P* = 0.0009; fig. 2*B*). The microbiome change was progressive. Sponges exposed to 3 weeks of shading showed microbiomes similar to control samples. In contrast, sponges exposed to 9 weeks of shading had microbiomes closer to those observed in shaded sponges (fig. 2*B*); sponge explants exposed to 6 weeks of shading had microbiomes intermediate between control and shaded sponges in NMDS space. Also, *L. chondrodes* explants exposed to scattered light (fig. 2*E*) with photosynthetic active radiation (PAR) below detection levels showed similar microbiome changes (ANOSIM *R*_1000_ = −0.1676, *P* = 0.9151) to those observed in shaded specimens (fig. 2*D*) but never lost their purple coloration.

**Fig. 3.**
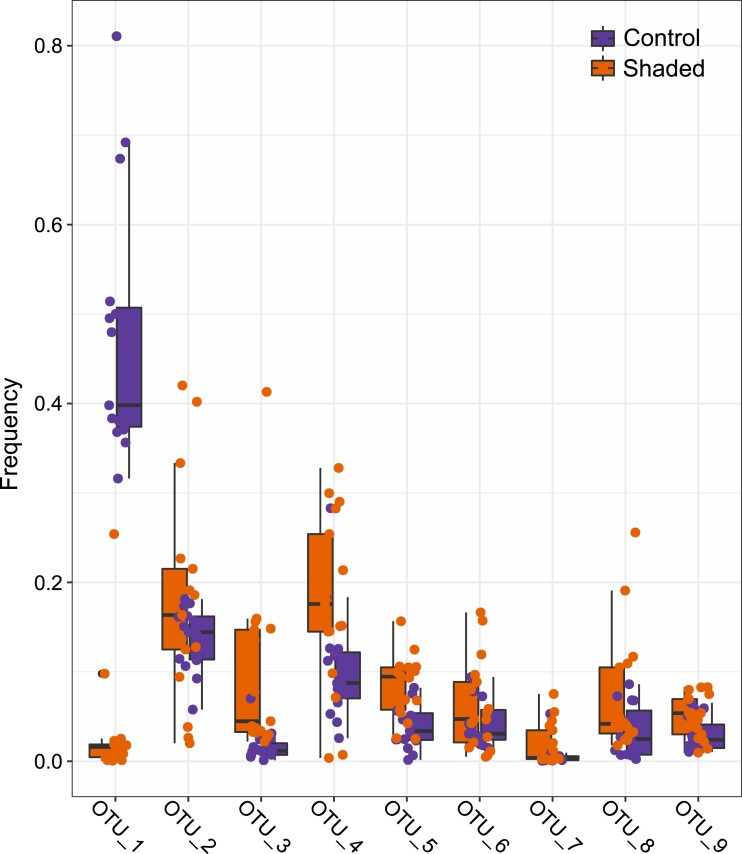
Abundance changes in *L. chondrodes*’ top nine core OTU in control versus shaded sponges. Note the generalized decay in the abundance of OTU_1 (Cyanobacteria) and the less clear but significant increase in the abundance of OTU_3 (Acidobacteria) and OTU_5 (Proteobacteria) in shaded compared to control sponges.

In general, the community shift observed in shaded *L. chondrodes* explants was driven by the collapse of the cyanobacterial population. This bacterial group was significantly depleted (Log2 Fold Change [LFC] = −4.60; *t*_18.40_ = −10.97, Bonferroni-corrected *P* < 0.001) in thread-like, shaded sponges compared to foliose, light-exposed sponges (fig. 3 and supplementary fig. S2, Supplementary Material online). In contrast, the abundance of other OTUs in *L. chondrodes*’ core microbiome (sensu; Vargas et al. 2021) barely differed between foliose and thread-like sponges, with only an acidobacterium (OTU_3) and a proteobacterium (OTU_5) increasing their abundance (LFC = 1.95 and 0.69, respectively; *t*_17.08_ = 3.18, Bonferroni-corrected *P*-value = 0.049 and *t*_29.81_ = 3.82, Bonferroni-corrected *P* = 0.006; fig. 3 and supplementary fig. S2, Supplementary Material online). Notably, the community changes observed upon shading were consistent among samples as judged by the correlation in the abundance ranks of the core OTUs measured in control and shaded samples (supplementary fig. S3, Supplementary Material online). This analysis revealed a significant correlation in the abundance ranks of the core OTUs between samples belonging to either the control or the shaded group but no significant correlation in the abundance ranks of core OTUs between control and shaded samples (supplementary fig. S3, Supplementary Material online). Clear changes in rank abundance could be observed for Cyanobacteria (OTU_1), which consistently occupied the first abundance rank in control samples but dropped to the eighth and ninth abundance rank in shaded sponges (supplementary fig. S3, Supplementary Material online). Acidobacteria (OTU_3) and one proteobacterium (OTU_5) also had marked rank abundance changes. Acidobacteria frequently occupied the seventh and eighth abundance ranks in control sponges, whereas in shaded samples, it occupied upper abundance rank levels (i.e., ranks one to eight; supplementary fig. S3, Supplementary Material online). The change in abundance rank of the proteobacterium (OTU_5) in shaded samples was less clear. This OTU occupied a broader range of abundance ranks in control and shaded samples. However, in shaded sponges, it occupied upper abundance ranks (i.e., rank three; supplementary fig. S3, Supplementary Material online) more frequently. All remaining OTUs either preserved their abundance rank or moved only one abundance rank in shaded compared to control samples.

### Shading Impairs Growth and Triggers a Body-Plan Reorganization in *L. chondrodes*

**Fig. 4.**
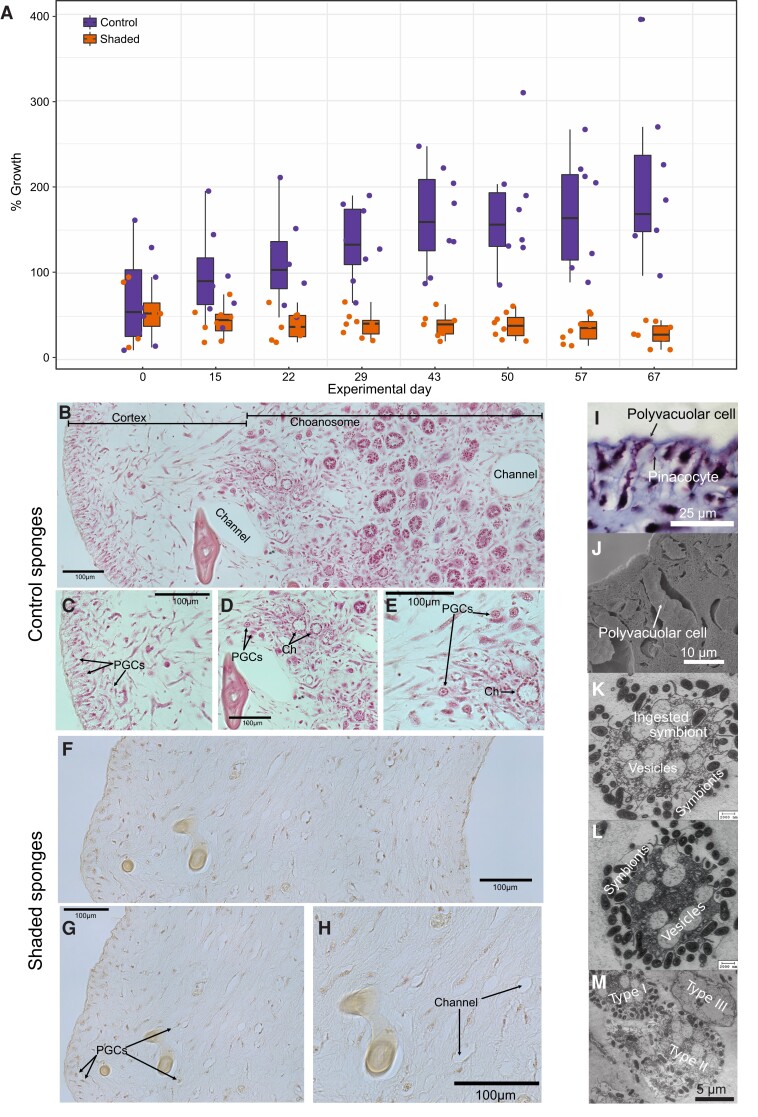
Growth and general microanatomy of control and shaded *L. chondrodes* specimens. (*A*) Growth of control and shaded *L. chondrodes* explants. (*B*) General microanatomy of control *L. chondrodes.* Note the highly organized cortex/choanosome in the control specimens, with a palisade of polyvacuolar gland-like cells (PGCs) beneath the pinacoderm (*C*), with well-developed choanocyte chambers (*D* and *E*), and the presence of multiple types of PGCs in the mesohyl (*C–E*). (*F*) Microanatomy of shaded, thread-like *L. chondrodes*. Note the unstructured appearance of the mesohyl, mainly composed of dispersed cells without a clear choanosome or palisade of PGCs (*F–H*). (*I*) Light and (*J*) scanning electron microscope images of *L. chondrodes* palisade of polyvacuolar gland-like cells and transmission electron microscopy images of these cells interacting with symbiont in the sponge mesohyl (*K–M*).

Shaded *L. chondrodes* explants only increased their area by ∼15%, whereas the area of control sponges increased by ∼150% after 67 experimental days (Curdt et al. 2022; fig. 4). A transition from a foliose to a thread-like morphology consistently accompanied growth stagnation and bleaching in *L. chondrodes* (see fig. 2*C*–*E*). This transition occurs without any evidence of tissue necrosis or degeneration and cannot be explained by light deprivation alone since sponges exposed to scattered light with PAR below detection levels also showed reduced growth and developed a thread-like morphology while retaining their color (see fig. 2*E*). To study the effect of shading on sponge morphology, we investigated the microanatomy of control and shaded sponges. Histological sections of control and shaded sponges revealed a contrasting micromorphology in these two treatment groups. Control sponges are highly organized, with a clearly delimited choanosome and a palisade of vacuole-rich cells (hereafter polyvacuolar gland-like cells [PGCs]) underneath the pinacoderm (fig. 4*B*, *I*, and *J*). Under control conditions, PGCs occur interspersed in the pinacoderm and form pore openings between exopinacocytes (fig. 4*I* and *J*). Notably, PGCs physically interact with and ingest bacteria in the mesohyl (fig. 4*K* and *L*), where three PGC morphotypes with varying vesicle sizes (fig. 4M) coexist. In contrast, shaded sponges had a disorganized micromorphology with no identifiable choanosome and a depletion of PGCs as judged by the reduction of *L. chondrodes’* characteristic subpinacodermal cell palisade (fig. 4*F–H*). Contrary to control sponges, shaded sponges did not have visible osculae, and choanocyte chambers were observed only rarely.

### *L. chondrodes* Regulates Immune and Developmental Coexpression Modules During the Foliose to Thread-Like Morphology Transition

To evaluate whether the morphogenetic changes observed in shaded *L. chondrodes* are accompanied by changes in the sponges’ transcriptional state, we used RNA-seq to de novo assemble a reference transcriptome for *L. chondrodes*. The assembly consisted of 128,686 transcripts >200 bp (N50 = 1,281 bp, mean length = 803 bp) and is highly complete (supplementary Table S1, Supplementary Material online). Upon translation, 37.75% of all the transcripts resulted in peptides >100 amino acids, of which 29.44% had a Swiss-Prot Blast match, and 36.21% matched a protein present in the marine sponge *A. queenslandica* (Fernandez-Valverde et al. 2015).

**Fig. 5.**
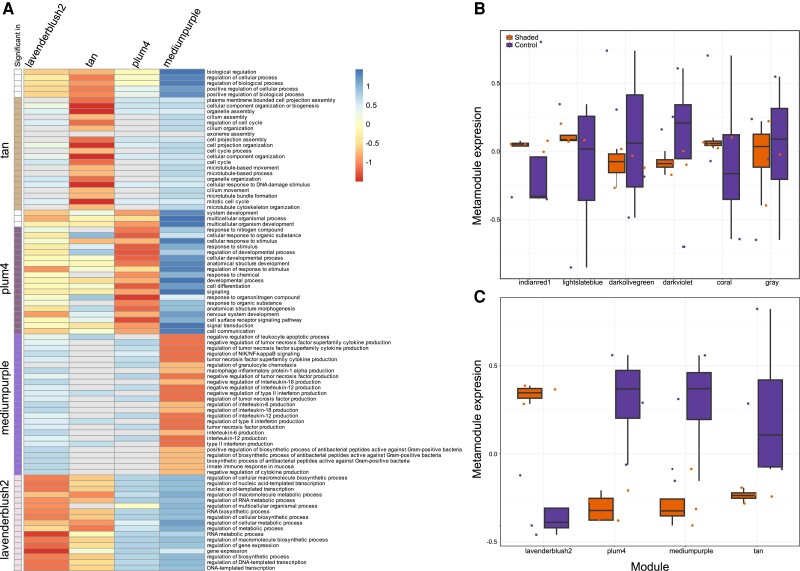
(*A*) Heatplot showing the 25 most significant GO terms enriched in metamodules showing significantly different eigenexpression patterns in control versus shaded *L. chondrodes*. Warm colors indicate high significance of a GO term enrichment analysis done for a particular module; the scale was constructed using the logarithm of the *P*-values obtained from TopGO. GO terms are grouped by metamodule. (*B*) Eigenexpression of metamodules not significant between control and shaded *L. chondrodes.* (*C*) Metamodules showing a significant eigenexpression difference in control versus shaded *L. chondrodes.* In *B* and *C*, color names represent specific coexpression modules as inferred by *wcgna*.

A comparison of the global expression patterns of shaded versus control samples revealed significant changes (Adonis Pseudo-*F*_1,7_ = 5.1164, *P* = 0.04) in the transcriptional state of the shaded sponges. A gene coexpression network analysis resulted in ten metamodules with 103 to 5,281 transcripts (fig. 5). Four metamodules had significantly different eigengene expression values in control versus shaded sponges (fig. 5). These metamodules contain ∼72% (12,943) of all the transcripts analyzed (17,893) and include the three most populated metamodules found with 5,281 (“lavenderblush2”), 4,595 (“tan”), and 3,114 (“plum4”) transcripts each. The largest significant metamodule (“lavenderblush2”) includes transcripts showing higher eigengene expression values in shaded than control sponges. This metamodule was enriched in transcripts involved in regulating transcription and gene expression, of macromolecule biosynthetic and metabolic processes, among others (fig. 5 and supplementary table S2, Supplementary Material online). The remaining significant metamodules had a higher eigengene expression in control sponges. Of these metamodules, the “plum4” metamodule was enriched in transcripts related to cellular and anatomical development, the regulation of signal transduction and cell communication, and the response to different stimuli. The “mediumpurple” metamodule grouped transcripts involved in the sponge's immune response, the regulation of interleukin and cytokine production, and the production of antibacterial peptides against Gram-positive bacteria. Finally, the “tan” metamodule was enriched in gene ontology (GO) terms related to the regulation of the cell cycle, of cilium and organelle assembly, and response to DNA damage (fig. 5, and supplementary tables S3–5, Supplementary Material online).

### Specific Developmental and Immune Pathways Are Differentially Modulated in Foliose Versus Thread-Like, Shaded Sponges

**Fig. 6.**
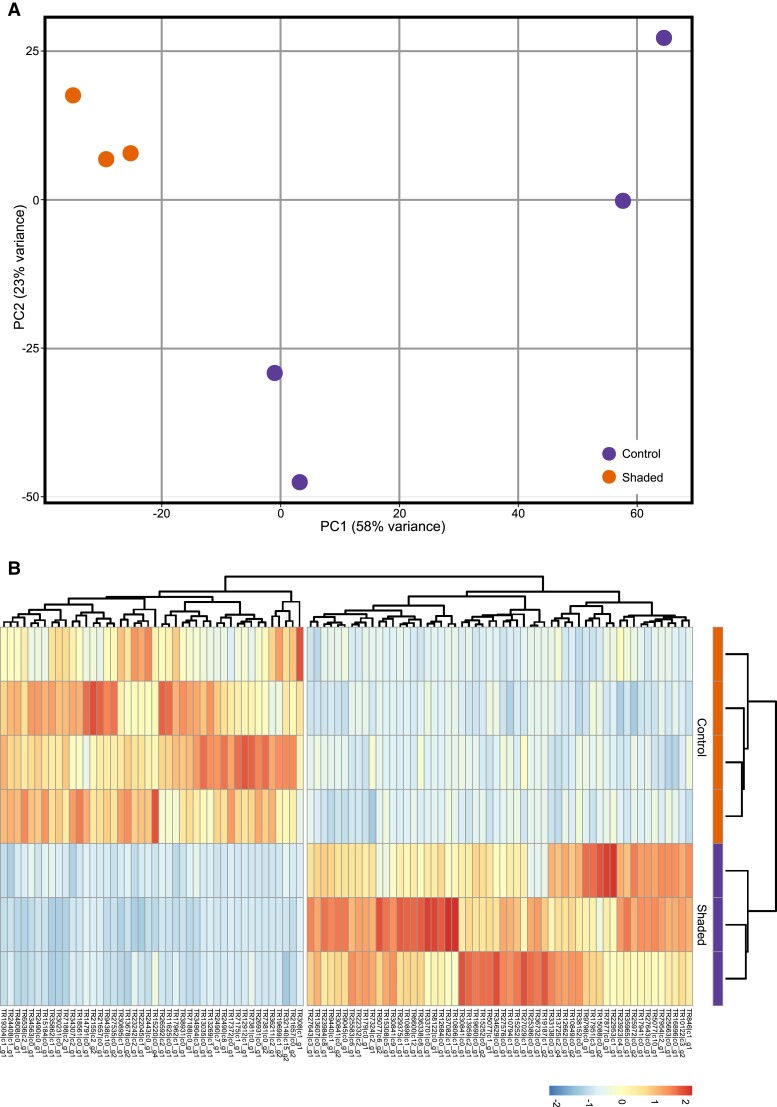
(*A*) Principal Component Analysis (PCA) of control and shaded *L. chondrodes* samples showing whole transcriptome differences in expression between the two groups. (*B)* Normalized (*z*-scored) counts of the top 100 more variable transcripts showing a marked difference in the gene expression in control versus shaded *L. chondrodes*.

**Fig. 7.**
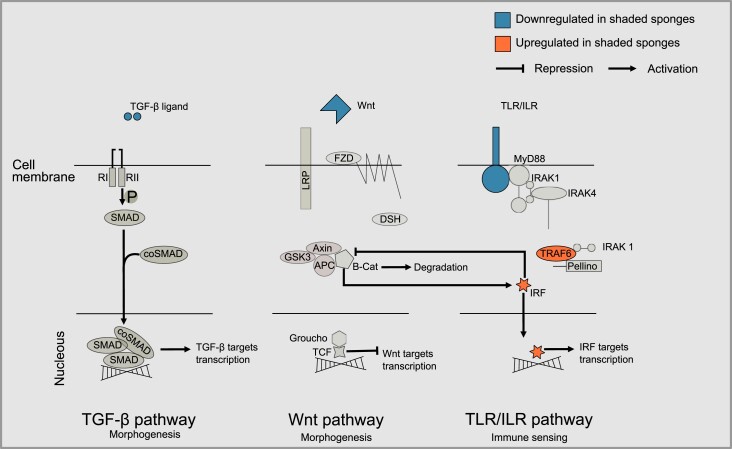
Upregulated and downregulated components of selected morphogenetic and immune pathways identified in control versus shaded, thread-like *L. chondrodes*. These pathways were chosen based on published studies showing their importance during development in sponges.

Gene expression was markedly different in shaded *L. chondrodes* explants (fig. 6). Between treatments, a total of 5,508 transcripts were differentially expressed (Benjamini-Hochberg-corrected *P* < 0.01). Of these, 1,166 transcripts were upregulated (LFC ≥ 1) in shaded sponges compared to the control, and 2,563 transcripts were downregulated (LFC ≤ −1). In agreement with our gene coexpression network analysis, GO term enrichment analyses of this set of genes also pointed to the regulation of canonical and noncanonical Wnt signaling and, generally, to processes linked to cell development and cell movement among differentially expressed genes (supplementary Table S6, Supplementary Material online). We found an ortholog of *A. queenslandica*'s WntA and a Wnt-like transcript of undetermined phylogenetic affinity (fig. 7 and supplementary fig. S4, Supplementary Material online) significantly underexpressed in shaded sponges. In addition to Wnt, we found a formin-bearing transcript related to *D*. *melanogaster*'s MWH protein (supplementary fig. S5, Supplementary Material online), a component of the noncanonical planar cell polarity (PCP) Wnt pathway, underexpressed in shaded sponges. Finally, shaded sponges also downregulated a putative transforming growth factor *β* (TGF-*β*) transcript and a putative ortholog of *Amphimedon*'s TLR receptor 1 (AmqIgTIR1) and upregulated a TNF receptor-associated factor (TRAF) and an ortholog of *A. queenslandica*'s interferon regulatory factor 1 (IRF1; fig. 7 and supplementary figs. S6–9, Supplementary Material online).

## Discussion

Broad, coupled morphological and transcriptomic changes correlated with the loss of a key symbiont have never been reported in sponges. In other animals, changes in the microbiome correlate with behavioral and developmental alterations (Cheesman et al. 2011; Shin et al. 2011; Sampson and Mazmanian 2015; Sharon et al. 2016; Klimovich et al. 2017). Our results in *L. chondrodes* provide evidence for a correlated response of the sponge host and its microbiome to environmental perturbations. The stark foliose to thread-like transition of the sponge body-plan parallels the microbiome changes caused by light deprivation and entails the modulation of diverse developmental, signaling, behavioral, and metabolic processes. It also correlates with the depletion of PGCs and the reduction in the number of choanocyte chambers and osculae in shaded versus control sponges. The reduction of the PGC population is particularly interesting as these cells physically interact with the microbiome in the mesohyl and can, therefore, mediate host–symbiont interactions in *L. chondrodes*.

It is important to note that light deprivation, a possible confounding factor in our experimental setting, cannot explain the sponge's observed morphogenetic/transcriptional response as sponges exposed to scattered light also transition to a thread-like morphology. As in light-deprived sponge explants, sponges exposed to scattered light also had a microbiome characterized by the loss of symbiotic cyanobacteria, indicating that the main driver of the morphogenetic changes observed in our study was the collapse of the photosymbiont population. The fact that other *L. chondrodes* core OTUs did not markedly respond to the shading treatment and the collapse of the cyanobacterial population further supports this interpretation and indicates that the core microbiome of *L. chondrodes* is resilient to compositional perturbations caused environmental changes, as reported in other sponges (Britstein et al. 2020). Notably, transplantation experiments on keratose sponges only resulted in morphological changes in sponges depleted in cyanobacteria in low-light availability environments, indicating that the lack of photosymbionts triggered the morphogenetic accommodation of the transplanted specimens, in agreement with our results (Maldonado and Young 1998). In conjunction, these observations and the results of our manipulative experiments provide strong evidence for an active reaction of the sponges that correlates with the decay in photosymbiont abundance.

Although the precise causal mechanism coupling microbiome changes with the observed reaction by the sponge host remains to be determined, our results suggest that both direct “immune” and indirect “sensing” via the perturbation in the sponge's carbon budget caused by a smaller, photosynthetically compromised cyanobacterial population, are active in *L. chondrodes*. In this regard, we observed an active regulation of metabolism-related GO terms in *L. chondrodes*. Coupled with the observation that, in agreement with previous field experiments on different cyanosponges (Thacker 2005), shading causes growth to stagnate and impairs choanocyte chamber development in *L. chondrodes*, the observed active change in metabolic regulation in shaded versus control sponges likely represents a compensatory reaction of the sponge to the reduction in photosymbiont-derived carbon upon shading (Maldonado and Young 1998; Hudspith et al. 2022). Supporting this interpretation, we found metamodules enriched in GO terms involved in the sponges’ response to oxygen levels, oxidative stress, and reactive oxygen species as expected if the sponges increased their metabolic rate upon symbiont depletion and, consequently, had a higher oxygen demand (supplementary Table S2, Supplementary Material online). Partial and complete microbiome removal causes a starvation-like response and developmental arrestment in the cockroach *Periplaneta americana* (de León et al. 2021). Our results in a sponge, an early-branching metazoan, highlight the importance of the microbiome for animal nutrition and development in general and point to an ancient co-evolutionary history of animals and their bacterial associates.

Direct “immune” recognition of the symbiotic bacteria, for instance, by PGCs, could be in place in *L. chondrodes* and generally in sponges (Yuen et al. 2014; Degnan 2015; Reynolds and Thomas 2016). Bleaching and reinfection with *Symbiodinium* modulate immune processes in the sponge *Cliona varians* (Riesgo et al. 2014), and sponges exposed to different microbial-associated molecular patterns activate genes associated with immune responses and animal–microbe interactions (Pita et al. 2018). In agreement with these findings, our results also revealed the modulation of transcription modules enriched in immune processes during the microbiome/morphogenetic transition observed in shaded *L. chondrodes*. Remarkably, we also found transcripts involved in developmental processes enriched in differentially expressed metamodules, pointing to a possible interaction between immune sensing and morphogenesis in *L. chondrodes*. In this regard, the modulation of specific elements of the Wnt, the TGF-*β*, the TLR, and the IRF pathways observed in *L. chondrodes* appears to be of particular interest as these pathways interact in different animal systems.

For instance, TLR signaling plays roles in development and innate immunity in the starlet sea anemone (Brennan et al. 2017) and the demosponge *A. queenslandica* (Gauthier et al. 2010). *Amphimedon* dynamically expresses several TLR signaling pathway elements during embryogenesis and in larval sensory cells (Gauthier et al. 2010). In *Hydra*, Myd88, an adaptor of the TLR signaling pathway, plays a role in bacterial sensing and mediates bacterial recolonization upon microbiome loss (Franzenburg et al. 2012). This same adaptor is involved in zebrafish in the bacterial modulation of the developmental Wnt pathway providing a mechanism whereby bacterial immune sensing and animal morphogenesis could be coupled in metazoans in general (Cheesman et al. 2011). Similarly, immune-modulating IRFs crosstalk with Wnt during the differentiation of immune cells from hematopoietic stem cells (HSCs) to produce different cell types in a dosage-dependent manner in animals (Luis et al. 2011; Scheller et al. 2013; Cohen et al. 2015). In mice, a regulatory circuit involving Wnt and Irf8, a *β*-catenin target gene that regulates *β*-catenin nuclear accumulation, exists, mechanistically coupling TLR and Wnt signaling during HSC differentiation (Scheller et al. 2013).

Wnt ligands activate at least three signaling cascades involved in different animal morphogenetic processes (Komiya and Habas 2008). In sponges, Wnt signalling plays a role in the development of the aquiferous system in a homoscleromorph (Lapébie et al. 2009) and a freshwater demosponge (Windsor and Leys 2010; Schenkelaars et al. 2016) and is expressed in parts of the aquiferous system and around osculae of the calcareous sponge *Sycon ciliatum* (Adamska et al. 2007; Leininger et al. 2014) and the demosponge *Halisarca dujardini* (Borisenko et al. 2016). Wnt ligands are also prominently expressed in the posterior poles of the larvae in *A. queenslandica* (Adamska et al. 2007), *H. dujardini* (Borisenko et al. 2016), and *S. ciliatum* (Leininger et al. 2014). In *S. ciliatum*, TGF-*β* ligands are coexpressed with Wnt around osculae (Leininger et al. 2014). TGF-*β*, an important regulatory element of the immune response, is involved in multiple developmental processes, including establishing the animal body axes (Travis and Sheppard 2014), and its expression is highly dynamic during development in *A. queenslandica* (Adamska et al. 2007) and *S. ciliatum* (Leininger et al. 2014). It is worth noting that the observation of reduced or absent choanocyte chambers and osculae in shaded *L. chondrodes* may indicate a similar role of Wnt in this sponge species that prompts further research. It also points to a possible developmental impairment in shaded *L. chondrodes*, suggesting that the regulatory circuits coupling immune TLR/IRF and morphogenetic Wnt signaling present in other animals also exist in sponges. Further research is needed to determine how sponge cells interact with their microbiomes and how these interactions affect the sponges’ transcriptional and developmental regulation.

Our results in *L. chondrodes* provide evidence of a link between the sponge's body-plan and metabolism and the state of its microbiome, implying that sponges can directly or indirectly detect and actively respond to changes in it. The co-regulation of transcripts involved in pattern recognition via TLR signaling (e.g., AmqIgTIR1 and IRF orthologs) and in morphogenesis, through the modulation of Wnt and TGF-*β* signaling, offers a possible molecular mechanism whereby sensing of symbionts by the sponge innate immune system can be coupled with Wnt- and TGF-*β*-mediated cell differentiation and cell motility to actively and dynamically reorganize the sponge's morphology and growth to avoid environmental conditions (e.g., shading) unfavorable for its microbiome. In addition, the necessary metabolic accommodation of the host to compensate for the loss of an important carbon source (i.e., the cyanobacterial population) can lead to the transcriptional modulation of cellular processes to cope with, for instance, a higher oxygen demand. In conjunction, our results suggest a deep evolutionary origin of the molecular crosstalk mechanisms used by animals to interact with their microbiomes. They also suggest that universal metazoan signaling pathways (e.g., Wnt, IRF, TLR, and TGF-*β*) may mediate host–symbiont interactions in sponges as it has already been shown in other animal groups (Cheesman et al. 2011; Franzenburg et al. 2012; e.g., Brennan et al. 2017; Daniel et al. 2017; Tang et al. 2017) implying that the genetic toolkit necessary to support complex microbiomes was already present in the last common ancestor of animals.

## Methods and Methods

### Description of the Aquarium System

Animals were kept in a 642-L marine aquarium under a 12-h day/12-h night cycle and controlled temperature and pH. Based on hourly measurements over one year (2017), average water temperature and pH were 24.92 ± 0.24 °C and 8.30 ± 0.14. Based on weekly measurements over one year (2017), the average PO_4_^−3^, NO_2_^−^, and NO_3_^−^ concentrations were 0.092 ± 0.071 mg/L, 0.014 ± 0.072 mg/L, and 2.681 ± 3.882 mg/L; the concentration of NH_3_/NH_4_^+^ was consistently below detection (i.e., <0.05 mg/L). These values were monitored hourly and weekly and were stable. Thus, they represented baseline values for the system. All experiments were carried out in a tank connected to the main 642-L aquarium tank through two water inlets and one central outlet that led the water back to the system water reservoir, from which is pumped back to the aquarium's main and experimental tanks. Water flowed constantly between the two systems, so sponge explants were exposed to the same temperature, pH, and nutrient measured in the main water tank. To avoid sediment accumulation on the experimental system, each inlet was connected to a sediment trap through which the water flowed before entering the experimental area of the tank. The water in the experimental tank then flows to the main central water outlet. The experimental system was arranged symmetrically to avoid confounding factors like water flow. A graphical description of the aquarium and experimental system can be found in supplementary figure S1, Supplementary Material online.

### Shading Experiments

Six sponge explants (1 cm in diameter) were exposed to shading in the same aquarium system where the parental sponges were kept. Shading was achieved using a black plastic foil mounted on top of polymethylmethacrylate panels covering the aquarium space where the explants were placed. Neither the plastic foil nor the polymethylmethacrylate panels were in contact with the water or blocked the water movement; that is, they were placed on top of the aquarium, allowing water to move in the system from the water inlets to the central outlet and only blocking the light.

Control and treatment explants were placed on (open) Petri dishes, separated to avoid contact (and fusion), and visually inspected every week. The Petri dishes were manually cleaned using a Pasteur pipette if sand or fine sediment accumulated. This process was done using light and generally took <1 h. The sponges were covered (if necessary) again after each check/cleaning. Due to the symmetrical arrangement of the water inflow in the experimental tank and the presence of a single, central outflow, both control and treatment sponges were exposed to similar conditions in terms of temperature, nutrients, water pH, dissolved oxygen, and exposure to water currents thus allowing us to eliminate potential confounding effects by these variables from the experiment. Treatment sponge explants were completely shaded by the black plastic foil or were allowed to receive only scattered light (i.e., PAR levels below detection). To achieve this, sponge explants were positioned in the marginal areas of the shaded aquarium section where direct light from the top is blocked, but the light from the illuminated aquarium section still diagonally penetrates the shaded area. The scattered light treatment allowed us to constrain the possible confounding effect light deprivation may have on the sponges.

We used temporal blocks to avoid pseudo-replication. For this, we repeated the shading experiment several times during a 2-year period sampling one explant at experimental time zero (control explant) and after 3, 5, 9, and 12 weeks of exposure to shading. Sampling was done by transferring the sponge explants from the aquarium to 8-mL vials filled with liquid nitrogen. To avoid transferring other organisms commonly found in the aquarium, like small amphipods or polychaetes, the sponge explants were visually inspected, carefully cleaned with a Pasteur pipette, and transferred to the liquid nitrogen-containing vial. Tissue samples for RNA extraction were kept at −80 °C. Sponge samples for microbiome analysis were fixed in 96% ethanol and kept at −20 °C until further processing. Thus, the data sets generated for this study used control and shaded explants from different temporal blocks that can be treated as replicates for statistical purposes. Finally, we also sampled control and shaded (white/bleached) regions naturally occurring in the stock sponge to assess whether the artificial shading treatment triggers similar microbiome community changes to those observed in shaded sections of *L. chondrodes*.

### 16S V4 Amplicon Sequencing and Evaluation of Community Composition Changes in Response to Bleaching

To characterize changes in the composition of *L. chondrodes*’ microbiome upon exposure to shading and loss of cyanobacterial symbionts, samples were extracted using the Macherey–Nagel NucleoSpin Tissue DNA extraction kit and used to amplify the V4 region of the 16S rRNA gene using indexed primers 515F and 806R (see Pichler et al. 2018). Amplicons were 150-bp pair-end sequenced on an Illumina MiniSeq. The resulting reads were processed using *vsearch* (Rognes et al. 2016) to define operational taxonomic units (OTUs) using a cutoff distance of 97% similarity and to obtain a sample by OTU abundance matrix. We chose this method over more advanced strategies (e.g., DADA2; Callahan et al. 2016) for backward compatibility with previous studies of this system (cf. Vargas et al. 2021), allowing us to identify the OTUs previously determined as part of the *L. chondrodes* core community. Community composition was analyzed in R 4.2.1 (R Development Core Team 2008) using the package *vegan* (Oksanen et al. 2017). We used an NMDS with the Bray–Curtis distance to visualize the similarity of the samples’ bacterial communities and an ANOSIM to test the hypothesis of no effect of the experimental treatments (i.e., direct shading vs. scattered light, and shading + scattered light vs. control condition) on the composition of the sponge microbiome. Prior to all analyses, we visually inspected the samples’ rarefaction curves to assess their comparability and conducted all analyses using rarefied samples. Since the results obtained from the rarefied samples were similar to those obtained from the original data set we continued using the nonrarified data set for further analyses. In addition, for the nine most abundant core OTUs (as defined in Vargas et al. 2021), we tested for significant changes in OTU frequency in control versus shaded sponges using Hotelling's *T*-Square test. This test was followed by individual, Bonferroni-corrected *t*-tests to identify individual OTUs significantly changing its relative abundance between treatments. To assess whether *L. chondrodes*’ top nine core OTUs changed their abundance ranks consistently in shaded versus control samples, we calculated Kendall's rank correlation coefficient between sample pairs and tested for significant (Bonferroni-corrected) rank correlations between samples. The Sample by OTU matrix and a bash script with the *vsearch* pipeline used to generate OTUs are available in the project repository. The R scripts used to analyze the data are also available in this repository.

### Histological Work

For histology, samples were fixed in a ∼4% formalin in seawater solution overnight at ∼5 °C, in 2.5% glutaraldehyde + 2 m sucrose in seawater or using Bouin's solution + 2 m sucrose. The tissue was dehydrated in a graded ethanol series and embedded in paraffin. Sections (5–15 μm thick) were made and stained using H&E or Masson trichrome. Documentation of the sections was performed on a BH-2 light microscope (Olympus) in combination with an Axio Cam I (Carl Zeiss) or a Leica Thunder Imager.

For scanning and transmission electron microscopy, samples were fixed in a fixation cocktail containing 2% glutaraldehyde and 1% osmium tetroxide in sterile filtered seawater and dehydrated in a graded ethanol series. Samples for the scanning electronic microscope (SEM) were embedded in methacrylate. The final mixture was prepared according to Reimer (1967) and Stockem and Komnick (1970). From the hardened blocks, semi-thin sections were cut with an HM 360 Mikrotom (Microm International GmbH). These cut samples were disembedded using xylene and transferred into ethanol or acetone. The dehydrated samples were dried on an Emitech K850 Critical Point Dryer (Quorum Technologies) and gold coated on an Emitech K500 Sputter Coater (Quorum Technologies). Imaging was done using an ESEM XL30 scanning electron microscope (Philips). For ultrastructural studies, samples were embedded in Durcupan ACM (Sigma-Aldrich). Ultrathin sections of ∼50–70 nm were placed on Formvar-coated copper grids and contrasted with uranyl acetate and lead citrate, according to Reynolds (1963). Imaging was done on an EM 900 and CEM 902a transmission electron microscope (Carl Zeiss) with an accelerating voltage of 80 keV.

### Transcriptome Sequencing, Assembly, and Annotation

RNA was extracted from flash-frozen sponge explants using a standard CTAB protocol followed by an isolation/clean-up using ZR-Duet™ DNA/RNA MiniPrep (Zymo). The concentration and quality of the RNA extracts were controlled on a NanoDrop (to obtain 260/280 and 260/230 ratios) and a Bioanalyzer 2100. Extractions with RNA Intergrity Number > 8.5 were used to produce stranded libraries for RNA-seq at the EMBL Sequencing Core. These libraries were quality controlled on a Bioanalyzer 2100 and 50-bp PE sequenced on an Illumina HiSeq 2000, quality-controlled with FastQC, and used for de novo transcriptome assembly in Trinity version 2.0.6 with default settings (Grabherr et al. 2011). Transcriptome completeness was assessed using BUSCO (Simão et al. 2015). The assembly was annotated against Swiss-Prot, the *A. queenslandica* AQU2 protein set (Fernandez-Valverde et al. 2015) and the KEGG database (http://www.genome.jp/kegg/). For transcripts that could be annotated using the UniProt Swiss-Prot database, the GO terms of the best UniProt match were fetched and used. Protein domains were searched using PfamScan.pl. Transcripts were translated using the program TransDecoder version 2.0.1 (https://github.com/TransDecoder/TransDecoder/wiki). All annotations were compiled on a csv metatable. The assembly, the annotation files, the metatable, and several scripts used to conduct the analyses are available in the project repository.

### Differential Gene Expression Analyses and GO Term Enrichment Analyses

Upon assembly of the *L. chondrodes*’ reference transcriptome, the program RSEM version 1.2.28 (Li and Dewey 2011) was used to obtain counts per transcript for each library. The count matrix generated was analyzed with the R package *wgcna* (Langfelder and Horvath 2008) to determine clusters of transcripts with similar expression patterns (hereafter, transcription modules). We allowed for clusters with a minimum size of 30 transcripts and merged modules using a module eigengene expression dissimilarity threshold of 0.35. For the resulting modules, significant differences in eigengene expression between control and shaded sponges were assessed using permutation-based Student's *t*-tests. To characterize the transcripts grouped in metamodules showing significantly different eigengene expression in control versus shaded specimens, we conducted GO term enrichment analyses with *TopGO* (Alexa and Rahnenfuhrer 2016) using a Fisher test on nodes with at least five annotations. For each metamodule, we used the top 25 most significant GO terms to assign metamodules biological functions and visualized the distribution of enriched GO terms among significant coexpression metamodules using a heat-plot using the logarithm of the Fisher test-derived *P*-value to color-coded GO terms in the different analyzed modules. In addition, DESeq2 (Love et al. 2014) was used to identify specific transcripts that were differentially expressed (|LFC| ≥ 1, False Discovery Rate < 0.01) in control versus shaded sponges and corroborate the modulation of specific pathways in shaded versus control sponges. R scripts to conduct these analyses are provided in the project's repository.

### Read Archiving and Project Repository

All short reads generated for this study were deposited in the European Nucleotide Archive (ENA) under Bioproject PRJEB24503. In addition, the assembled and annotated reference transcriptome and all scripts and data matrices used in this study can be retrieved at https://gitlab.lrz.de/cbas/cbas_resources.

## Supplementary Material

msad138_Supplementary_Data

## Data Availability

All short reads generated for this were deposited in the ENA under Bioproject PRJEB24503. In addition, the assembled and annotated reference transcriptome and all scripts and data matrices used in this study can be retrieved at https://gitlab.lrz.de/cbas/cbas_resources.
