# Supplementary material for: Body-Plan Reorganization in a Sponge Correlates with Microbiome Change
Source: Mol Biol Evol. 2023 Jun 8;40(6):msad138. doi: 10.1093/molbev/msad138 (PMC10308213; doi:10.1093/molbev/msad138)
Supplement: msad138_Supplementary_Data [file msad138_supplementary_data.zip › Vargasetal_CBASShading_SupplementaryTable_01.pdf]

Supplementary Table 1. BUSCO results for the reference transcriptome of *Lendenfeldia chondrodes*.

**BUSCO category**	**Total found**	**Percentage**
Complete and Single Copy	579	59.2%
Complete and Duplicated	316	32.3%
Fragmented	38	3.9%
Missing	45	4.6%

Total BUSCOs = 978.
